# Di (2-ethylhexyl) Phthalate Exposure Impairs Growth of Antral Follicle in Mice

**DOI:** 10.1371/journal.pone.0148350

**Published:** 2016-02-04

**Authors:** Lan Li, Jing-Cai Liu, Fang-Nong Lai, Huan-Qi Liu, Xi-Feng Zhang, Paul W. Dyce, Wei Shen, Hong Chen

**Affiliations:** 1 College of Animal Science and Technology, Northwest A&F University, Yangling, 712100, China; 2 College of Life Sciences, Qingdao Agricultural University, Qingdao, 266109, China; 3 College of Animal Science and Technology, Institute of Reproductive Sciences, Key Laboratory of Animal Reproduction and Germplasm Enhancement in Universities of Shandong, Qingdao Agricultural University, Qingdao, 266109, China; 4 Department of Animal Sciences, Auburn University, Auburn, AL, 36849, United States of America; Institute of Zoology, Chinese Academy of Sciences, CHINA

## Abstract

Di (2-ethylhexyl) phthalate (DEHP) is a widely used plastic additive. As an environmental endocrine disruptor, it has been shown to be harmful to the mammalian reproductive system. Previous studies indicated that DEHP inhibited the development of mouse ovarian follicles. However, the mechanisms by which DEHP affects ovarian antral follicle development during the pre-puberty stage are poorly understand. Thus, we investigated the effects of direct DEHP exposure on antral follicle growth in pre-pubescent mice by use of intraperitoneal injection. Our results demonstrated that the percentage of large antral follicles was significantly reduced when mice were exposed to 20 or 40 μg/kg DEHP every 5 days from postnatal day 0 (0 dpp) to 15 dpp. In 20 dpp, we performed microarray of these ovaries. The microarray results indicated that mRNA levels of apoptosis related genes were increased. The mRNA levels of the apoptosis and cell proliferation (negative) related genes *Apoe*, *Agt*, *Glo1* and *Grina* were increased after DEHP exposure. DEHP induced the differential gene expression of *Hsp90ab1*, *Rhoa*, *Grina* and *Xdh* which may play an important role in this process. In addition, TUNEL staining and immunofluorescence showed that DEHP exposure significantly increased the number of TUNEL, Caspase3 and γH2AX positive ovarian somatic cells within the mouse ovaries. Flow cytometer analyses of redox-sensitive probes showed that DEHP caused the accumulation of reactive oxygen species. Moreover, the mRNA expression of ovarian somatic cell antioxidative enzymes was down-regulated both *in vivo* and *in vitro*. In conclusion, our data here demonstrated that DEHP exposure induced oxidative stress and ovarian somatic cell apoptosis, and thus may impact antral follicle enlargement during the pre-pubertal stage in mice.

## Introduction

Phthalic acid esters (PAEs) are esterified derivatives of the phthalate acids which are widely added in the manufacturing of plastic [1]. Among the plasticizers, di (2-ethylhexyl) phthalate (DEHP) is one of the most common used [2]. DEHP is used to increase the flexibility of polyvinyl chloride (PVC) in consumer products, like building materials, maquillage, food containers, toys, and clothing [3]. Moreover, DEHP exists in medical products as well [3]. The estimated global annual DEHP output is between 2205 and 8818 billion pounds [4]. Due to the weak chemical binding of DEHP to plastic products, dissociated DEHP is widely present in the environment [5]. Humans are exposed to DEHP in direct and indirect ways, such as oral ingestion, inhalation, and dermal exposure [5]. Ingestion of DEHP is mainly absorbed into the blood circulatory system through the intestines and lungs [6]. DEHP has shown hepatotoxicity, reproductive toxicity, and developmental toxicity in experimental animals [6]. From previous studies, DEHP has been associated with hepatic, renal, testicular, ovarian, and other tissue diseases [7–10]. Thus, the health risks of DEHP exposure to humans has attracted substantial attention.

The ovary is the primary reproductive organ in the female [11]. It regulates many aspects of female endocrinology and provides a microenvironment essential for germ cell development [12]. The health of the ovary is of vital importance because it is the site of female gamete production, and it is also responsible for the secretion of certain sex hormones [13]. Functional disruption of the ovary may lead to many reproductive complications such as anovulation, abnormal estrogen secretion, premature ovarian failure, and even infertility [14–19]. As an endocrine-disruptor chemical DEHP and its derivative mono (2-ethylhexyl) phthalate (MEHP) lead to polyovular follicles, accelerated depletion of the primordial follicle pools, and a decrease of sex steroid hormone production [20–22]. From ours and others previous studies DEHP impacted oocyte maturation and changed the DNA methylation status of imprinted genes, moreover, these kind of modifications could be inherited to the germ cells of offspring [23–26]. Therefore, DEHP exposure could impair ovarian development across generations [25].

We previously demonstrated that, bisphenol A (BPA), one of an exogenous endocrine disruptor impacted primordial follicle assembly and altered the DNA methylation status of mouse oocytes [27, 28]. As an endocrine disruptor and reproductive toxicant, DEHP have ambivalent effects on different developmental stages of ovarian follicles. Recent study have shown that newborn mice exposed to DEHP showed an inhibition of oocyte nest breakdown and primordial follicle assembly, and these kind of impairments were mediated by estrogen receptors (ERs) [29]. Hannon and his colleagues found that MEHP accelerated the transition of primordial follicles to primary follicles via over-activating the PI3K signaling pathway *in vitro* [22]. An *in vivo* study indicated that following DEHP exposure for 10 days, the recruitment of primordial follicles into the growing pool was accelerated in adult ovaries [20]. These results together indicate that DEHP severely affects folliculogenesis in mice. DEHP has also been shown to inhibit the proliferation of granulosa precursor cells during the process of folliculogenesis [29]. The *in vitro* culture of adult mouse ovarian antral follicles demonstrated that MEHP, but not of DEHP inhibits antral follicle growth, induces atresia, and inhibits steroidogenesis [22]. Interestingly, when co-treated with estradiol, it partly rescued the toxic effects of MEHP on antral follicles, which showed the induction of atresia and the expression changes of apoptosis genes [30].

While previous work has demonstrated that DEHP impairs mouse folliculogenesis, both *in vivo* and *in vitro*, the effects of DEHP on the growth of antral follicles remain largely unknown. We hypothesize that DEHP exposure may accelerate ovarian somatic cell apoptosis during the pre-pubescent ovarian antral follicle growth. Therefore, we designed the present study to investigate the effects of DEHP exposure on antral follicle growth in pre-pubescent mouse ovaries. Further, we asked whether relative low doses of DEHP exposure are sufficient to inhibit antral follicle growth and investigated the potential causes underlying the effects of DEHP exposure on follicle development.

## Materials and Methods

### Animals and Experimental Design

CD1 mice (Vital River, Beijing, China) were used in all experiments. We used a total of 104 mice in this study. All mice were housed in temperature controlled (21–22°C) rooms and on a 12 h light, 12 h dark cycles (lights off at 19:30) with free access of food (Keaoxieli, Beijing, China) and water unless otherwise stated. We define the newborn mice as postnatal day 0 (0 dpp). All experimental animals in this research were reviewed and approved by the Ethics Committee of Qingdao Agricultural University (agreement No. 2014–07), and according to criteria outlined in the Guide for the Care and Use of Laboratory Animals published by the National Institutes of Health. To determine mouse health and body condition, we regularly checked the feed and water intake, coat and mental state of experimental mice at every 8 a.m. and 4 p.m. Humane endpoints were used during the animal survival study, with mice euthanized by CO_2_ inhalation upon signs of distress. All animals were sacrificed by decapitation at the end of the study.

DEHP was purchased from Sigma (36735-1G, USA). According to the safe assessment of the Food and Drug Administration (FDA) no observed adverse effect level (NOAEL) and lowest observed adverse effect level (LOAEL) of pregnant mice intraperitoneal is 4000 and 8000 mg/kg/day [31]. The experimental DEHP doses were far lower than the risk assessment dosage in accordance with FDA [31]. Stock solutions of DEHP were prepared using dimethylsulfoxide (DMSO) as the solvent in various concentrations (0.256 and 2.56 M) that allowed an equal volume to be added to culture wells for each treatment group to control for solvent concentration during the culturing of granulosa cells *in vitro*. Final concentrations in culture were 10 and 100 μM of DEHP. We chose these doses based on previous studies on the effects of DEHP exposure on cultured cells [25].

In view that, oral exposure of lactation mice may lead to a variation of offspring DEHP absorption. We performed an intraperitoneal injection (IP injection) method for direct DEHP exposure of neonatal female mice. However, neonatal mice may not tolerate daily DEHP IP injection and could result in neonatal death. So we performed DEHP IP injection for neonatal mice every five days. Newborn mice were injected with DEHP at doses of 0, 20, and 40 μg/kg body weight in 0.1% DMSO or 0.1% DMSO alone as a vehicle control at 5 dpp, 10 dpp and 15 dpp. At 20 dpp, the ovaries were collected for paraffin section and total RNA was extracted for gene chip analysis. We chose these doses based on our previous evidences that DEHP exposure leads to imprinted genes DNA methylation level changes in oocytes of mother and offspring [23]. In addition, IP injection of these doses of DEHP during the neonatal and juvenile to pre-pubertal stages in mice leads to premature ovarian failure symptoms [24].

### HE Staining

Ovaries from at least 3 mice per group of DEHP treatment were fixed in 4% paraformaldehyde (Solarbio, P1110, China) for 12 h. Ovaries were dehydrated and embedded in paraffin. Next, fixed ovaries were sectioned serially every 5 μm and attached mounted onto glass slides, heated at 60°C for 2 h, then applied for HE staining and immunofluorescence. After two steps of xylene dewaxing, slides were dehydrated in a graded ethanol series. Following by hematoxylin for 7 min, 1% Hydrochloric acid alcohol for 20 s, then put in water for 30 s. A series of graded ethanol/water solutions were then utilized and processed with eosin for 20 s, after two steps of xylene dewaxing, the slides were mounted with neutral resin. Pictures were taken under a BX51 microscope (Olympus, Japan).

### Immunofluorescence

After two steps of xylene dewaxing, slides were washed in a series of graded ethanol/water solutions. Then, slides were further incubated in 0.01 M sodium citrate at 96°C for 10 min. Samples were further blocked with BDT (3% BSA, 10% normal goat serum in TBS) for 45 min and incubated with rabbit anti-Caspase3 polyclonal antibody at a dilution of 1:150 (Abcam, ab2302, HongKong, China), rabbit anti-γH2AX polyclonal antibody at a dilution of 1:150 (Abcam, ab13840, HongKong, China), overnight at 4°C. After incubation, slides were washed three times with phosphate-buffered saline (PBS), then sections were incubated with CY3-conjugated goat anti-rabbit secondary antibody at a dilution of 1:150 (Beyotime, A0562, Nantong, China) at 37°C for 30 min. Vectashield (H-1000; Vector, Shanghai, China) was used to mount the slides. The proportion of Caspase3 and γH2AX positive cells was determined by calculating the number of positive cells in 5 different serial sections. All experiments were repeated at least three times independently.

### Follicle Counting

Technical limitations rejected accurate follicle number counting in histology sections. Our large and small antral follicle counting was based on the follicle counting method of Kim and his colleagues study with a few modifications [32]. Briefly, large antral follicles were defined as follicle with antrum and a diameter > 150 μm. The rest of antral follicles were regarded as small antral follicles. To compare the large and small antral follicle percentage per ovary, we sliced the whole ovary into numerous sections. We put the sections on glass slides in order. We counted the large and small antral follicles on the17^th^, 33^th^, 50^th^, 67^th^, and the 83^th^ slides to represent the large antral follicle intensity for the ovary. To compare the large and small antral follicles per ovary, the number of large follicles and small follicles per ovary was divided by the number of total follicles. Therefore, the “large and small antral follicle number” in this study represents a relative but not the actual follicle number per ovary.

### TUNEL Staining

Ovary apoptosis analysis was performed using the In Situ Cell Death Detection Kit (Roche, 12156792910, Germany). The ovary paraffin sections were heated at 60°C for 2 h then washed in xylene and rehydration through a series of ethanol and double distilled water. The sections were treated with proteinase K for 15 min at room temperature, and then wash three times with PBS. Then 50 μl of TUNEL reaction mixture (Enzyme Solution and Label Solution; 1:9) was added to the slides. For negative control, 50 μl label solution was added to the slides. Then the slides were incubated in a humidified atmosphere for 60 min at 37°C in the dark. The nuclei were stained with Hoechst33342 (Beyotime, C1022, China). TUNEL images were obtained under a fluorescence microscope (BX51; Olympus, Japan).

### RNA Extraction and Quantitative RT-PCR

Total RNA was extracted using the RNAprep pure Micro Kit (Aidlab, RN28, Beijing, China) according to the manufacturer’s instructions. Reverse transcription was performed using the TransScript^®^ One-Step gDNA Removal Kit and cDNA Synthesis Kit (TransGen Biotech, AT311, Beijing, China). All primers used in this research are listed in Table 1. Relative quantification analysis was carried out with the LightCycler 480 II (Roche, Germany) using the LightCycler^®^ 480 SYBR Green I Master Kit (Roche, 04887352001, Germany) according to the manufacturer’s instructions. Each sample contained 3 technical replicates and reactions were performed with 3 biological replicates. The PCR conditions were as follows: 10 min at 95°C, followed by 45 cycles of 95°C for 10 s, 60°C for 30 s and 72°C for 20 s. Gene expression levels were using *beta-actin* for normalization. Relative transcript abundance was calculated using the 2^-ᐃCT^ method [24]. Data was expressed as mean ± standard deviation (SD). and calculated from independent biological replicates at least three times.

**Table 1:** Primers used for RT-qPCR.

Genes	Sequences (5'-3')	Fragment size (bp)	Accession No.
***Apoe***	F:TGCTGTTGGTCACATTGCTG	160	NM_001305819.1
	R:TCTTCCTGGACCTGGTCAGA		
***Agt***	F:ATTCAGGGCTTGCTGGTCA	204	NM_007428.3
	R:CCTGTTGATTTTCTCAGTGGC		
***Glo1***	F:CCCTGCTATGAAGTTCTCGC	199	NM_001113560.1
	R:CCCAATGTGACCAAATCCAC		
***Grina***	F:GTCTGCTTCACGGTGGTCAT	241	NM_023168.3
	R:ATTCTTCTGGGCTCAGGGAC		
***Grem1***	F:TATCTGAAGCGAGATTGGTGC	144	NM_011824.4
	R:TTCCTCCTTTCGGATGTGC		
***Hsp90ab1***	F:TGACATCATCCCCAACCCT	188	NM_008302
	R:CCGAGTAGAATCCGACACCA		
***Rhoa***	F:TGGGAAGCAGGTAGAGTTGG	239	NM_016802.5
	R:GTCTCGTGTGCTCGTCATTC		
***Bcl-2***	F:GCAGAGATGTCCAGTCAG	127	NM_009741
	R:CACCGAACTCAAAGAAGG		
***Bax***	F: ATGCGTCCAAGGAAGACTGAG	162	NM_007527
	R: CCCCAGTTGAAGTTGCCATCAG		
***Caspase3***	F:GACTGGGATGAACCACGACCC	205	NM_001284409.1
	R:TCTGACTGGAAAGCCGAAAC		
***Caspase9***	F:CTGGGAAGGTGGAGTAGGAC	189	NM_015733.5
	R:GCGGTGGTGAGCAG		
***Cat***	F:CAGCGACCAGATGAAGCAGT	236	NM_009804.2
	R:CCTCAAAGTATCCAAAAGCACC		
***Gpx1***	F:GGAGAATGGCAAGAATGAAGAG	135	NM_008160.6
	R:AGGAAGGTAAAGAGCGGGTG		
***Sod1***	F:GGGTTCCACGTCCATCAGTA	128	NM_011434.1
	R:TTGCCCAGGTCTCCAACAT		
***β-actin***	F:TCGTGGGCCGCTCTAGGCAC	255	NM_007393.3
	R: TGGCCTTAGGGTTCAGGGGGG		

### Analysis of Microarray Profiling and Construction of Protein Interaction Networks

The protocols of total RNA microarray hybridization were consistent with our previous study [33]. Briefly, the integrity and concentration of total RNA were measured by the Agilent 2100 Bioanalyzer (Agilent Technologies, Santa Clara, CA, USA). We used 6 μg of high quality RNA labeled with Cy5, and hybridized to a mouse oligo microarray (Phalanx Mouse Whole Genome One Array^TM^; Phalanx Biotech Group, Palo Alto, CA, USA). Each array contained 26423 DNA oligonucleotide probes of the sense strand. After hybridization, the fluorescent signals on the array were scanned using an Axon 4000 (Molecular Devices, Sunnyvale, CA, USA). Data analysis was performed based on the manufacturer’s instructions. We used R program to build the volcano map and heatmap. The Differential Expression Genes (DEGs) were defined as log2 |fold change| > 1 (absolute |fold change| > 2) and P < 0.05. We screened the DEGs, we used DAVID (https://david.ncifcrf.gov/) for Gene Ontology enrichment analysis, we found a series of DEGs related to apoptotic process was clustered and string protein databases were used to construct the apoptotic related proteins interaction networks.

### Culturing of Mouse Ovarian Somatic Cells *In Vitro*

In order to simulate the 20 dpp ovarian development in an *in vitro* experiment, we used 13 dpp mouse ovaries and cultured ovarian somatic cells *in vitro* for 7 days. Briefly, 13 dpp female CD1 mice were euthanized and ovaries were isolated in 0.2% collagenase with watchmaker forceps to crush the ovaries. Then the collected primary follicles and early secondary follicles were digested by 0.25% trypsin-EDTA for 7 min at 37°C. After centrifuging for 4 min at 1200 rpm, supernatant was aspirated off and the isolated cells were cultured in ovarian somatic cell culture medium in a 6 cm adherent culture dish. The ovarian somatic cell culture was using Dulbecco’s modified Eagle’s medium/F12 (HyClone, SH30265.01, Beijing, China) supplemented with 10% fetal bovine serum (FBS; Gibco, 10099–141, USA), 0.23 mM sodium pyruvate (HyClone, SH40003-12, China), 100 IU/ml of penicillin G, and 100 mg/ml of streptomycin sulfate. In the process of ovarian somatic cell culture, 1.5 ml media was changed with fresh media once on the second day. In order to ensure the initial cell density remained constant, we performed DEHP exposure on passaged cells. After 4 days of culture (17 dpp), ovarian somatic cells were digested by 0.25% trypsin-EDTA incubation and then cultured with DEHP for 3 days (20 dpp). The concentration of DEHP used was 0, 10 and 100 μM. Media were changed once on the second day. After DEHP treatment for 3 days, ovarian somatic cells were collected for RNA extraction or flow cytometry analysis.

### Flow Cytometer Analyses

After 3 days culture with DEHP, cultured ovarian somatic cells were trypsinized and collected into a 1.5 ml Eppendorf tube. Incubated with serum-free mouse granulosa cell culture medium with 10 μM dichlorofluorescein diacetate (DCFH-DA, Beyotime, Nantong, China) or 5 μM dihydroethidium (DHE, Beyotime, Nantong, China) at 37°C for 30 min. After washing 3 times using serum-free mouse granulosa cells culture medium, cells were analyzed with a flow cytometer (BD FACSCalibur, FACS101, USA). From 5000 to 10000 events were acquired per sample. DCF fluorescence was detected at an excitation wavelength of 488 nm and an emission wavelength of 525 nm. DHE fluorescence was detected at an excitation wavelength of 535 nm and an emission wavelength of 610 nm. Quantification analysis was completed using ModFit software.

For fluorescence activated cells sorting (FACS) analysis of ovarian somatic cells for the *in vivo* experiments, 20 dpp ovaries were collected. Ovaries were minced using scissors. The ovary slurries were digested with collagenase (0.2%) for 20 min at room temperature. The cell suspensions were filtered through a 200 mesh cell strainer, and the filtrate suspension were further collected into a 1.5 ml Eppendorf tubes. The cell suspensions were centrifuged at 300 g for 5 min at room temperature, and then the supernatant carefully decanted. Resulting cell pellets were resuspended with 1 ml serum-free DMEM/F12 media. Cells were then incubated with serum-free mouse granulosa cell culture medium with 10 μM DCFH-DA at 37°C for 30 min. Then washed 3 times using serum-free mouse granulosa cell culture medium, and analyzed by flow cytometry (BD FACSCalibur, FACS101, USA). DCF fluorescence was detected at an excitation wavelength of 488 nm and an emission wavelength of 525 nm. Quantification analysis was completed using ModFit software.

### Statistical Methods

For each set of results, independent trials were repeated at least three times; data are represented as mean ± SD. Differences among groups were statistically tested by Student’s t-test or one-way analysis of variance (ANOVA) followed by the Tukey’ test for multiple comparisons using Graph-Pad Prism analysis software. Comparisons were considered significant at P < 0.05 (*) and extremely significant at P < 0.01 (**).

## Results

### Ovarian Morphological Changes in DEHP Exposure

**Fig 1:**
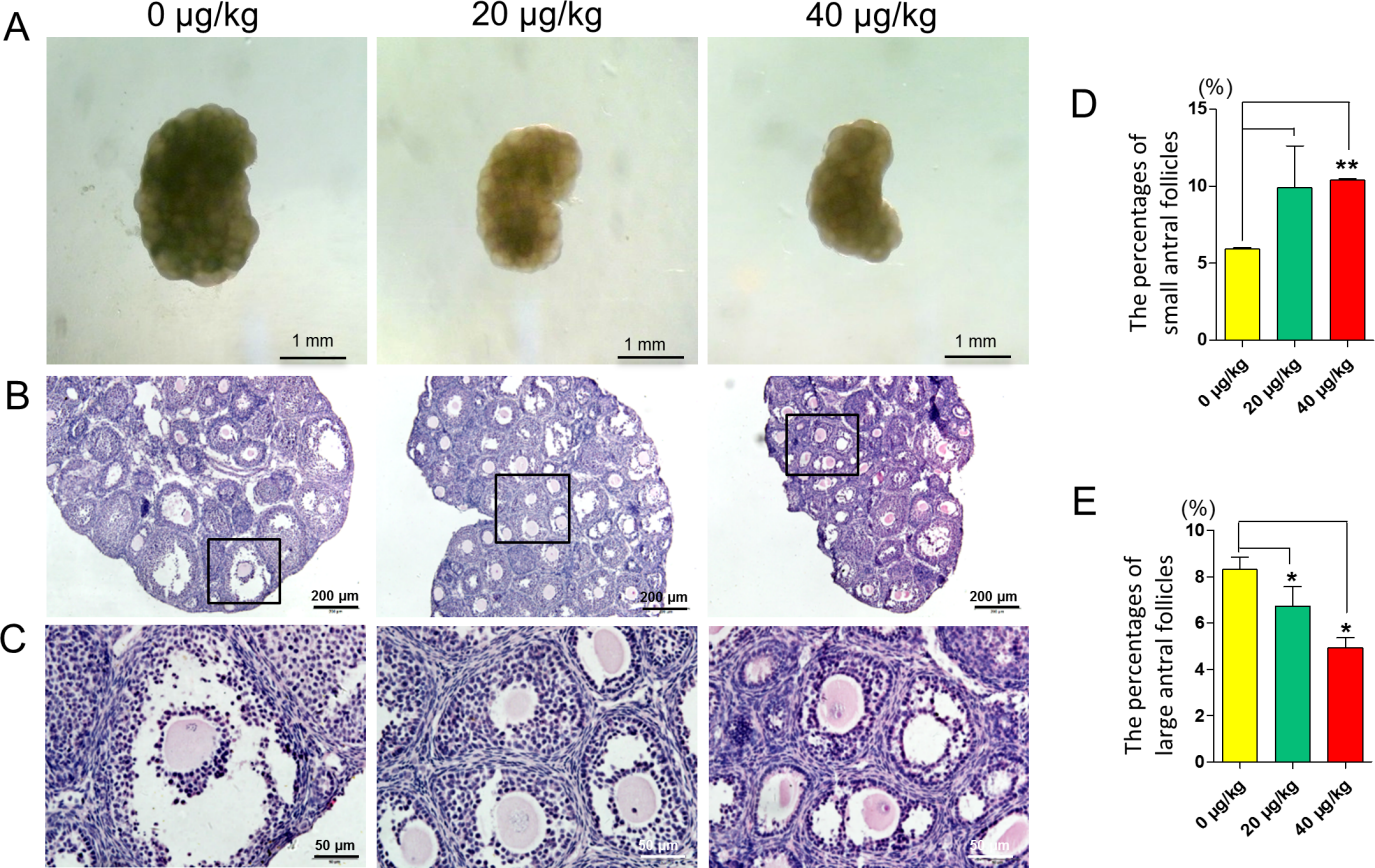
Effect of DEHP exposure on mouse ovaries. (A) Control and DEHP-treatment group of 20 dpp mouse ovarian morphology. (B-C) Control and DEHP-treatment groups of 20 dpp mouse ovarian HE histochemical sections. (D) The percentages of small antral follicles in the control and DEHP-treatment groups. (E) The percentages of large antral follicles in the control and DEHP-treatment groups. Compared to the control group the percentage of each group was presented as mean ± SD. All experiments were repeated at least three times independently. (* P < 0.05; ** P < 0.01).

To evaluate the effects of DEHP exposure on mouse ovarian development, during the pre-puberty period, newborn mice were injected intraperitoneally with DEHP at doses of 0, 20, and 40 μg/kg body weight at 5 dpp, 10 dpp and 15 dpp. At 20 dpp, we collected the ovaries for paraffin processing and sectioning. As showed in Fig 1A, the ovaries of DEHP treated groups were smaller in volume when compared with the control group. From the HE sectioning, we observed morphological changes of the ovarian follicles in the DEHP treatment groups, and the number of large antral follicle was reduced in DEHP treatment groups. (Fig 1B and 1C).

To investigate the effect of DEHP exposure on antral follicle development, we then counted each stage of ovarian follicles. The results show that, compared with control group, the percentage of small antral follicles (diameter < 150 μm) were significantly increased in 40 μg/kg DEHP-treatment groups (P < 0.01) (Fig 1D) and the percentage of large antral follicles were significantly decreased in the 20 μg/kg and 40 μg/kg DEHP-treated groups (P < 0.05) (Fig 1E).

### Microarray Data Analysis and Validation

**Fig 2:**
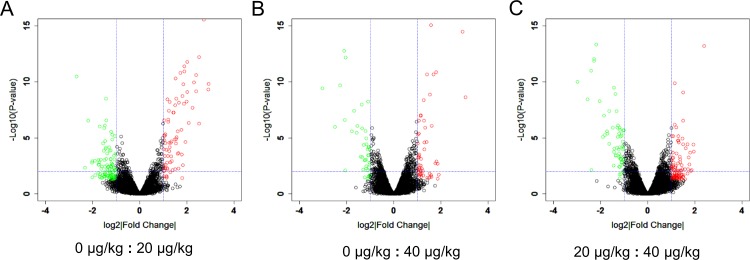
Scatterplot of DEHP treated ovarian gene expression profiling. Red plot represents genes up-regulated significantly and green plot represents genes down regulated significantly. (A) Control group *vs*. 20 μg/kg DEHP-treatment group, (B) Control group *vs*. 40 μg/kg DEHP-treatment group, (C) 20 μg/kg DEHP-treatment group *vs*. 40 μg/kg DEHP-treatment group.

Since DEHP exposure significantly reduced the percentage of large antral follicles, microarray was utilized to investigate the gene expression profiles following DEHP exposure. The volcano plot showed that, when compared with the control group, the expression of 78 and 54 genes were significantly up-regulated in the 20 μg/kg and 40 μg/kg DEHP-treatment groups, respectively (Fig 2A and 2B). Similarly, the expression of 102 and 43 genes were down-regulated in the 20 μg/kg and 40 μg/kg DEHP-treatment groups, respectively (Fig 2A and 2B). Compared with the 20 μg/kg DEHP-treatment group there were 101 genes showing mRNA expression that was up-regulated while in 65 genes expression was down-regulated in the 40 μg/kg DEHP-treatment group (Fig 2C). All these results show that low dose DEHP exposure can change the gene expression patterns in young mouse ovaries. In addition, DEHP exposure induced changes in the expression of premature ovarian genes and displayed a variation between the 20 μg/kg and 40 μg/kg DEHP-treatment groups.

**Fig 3:**
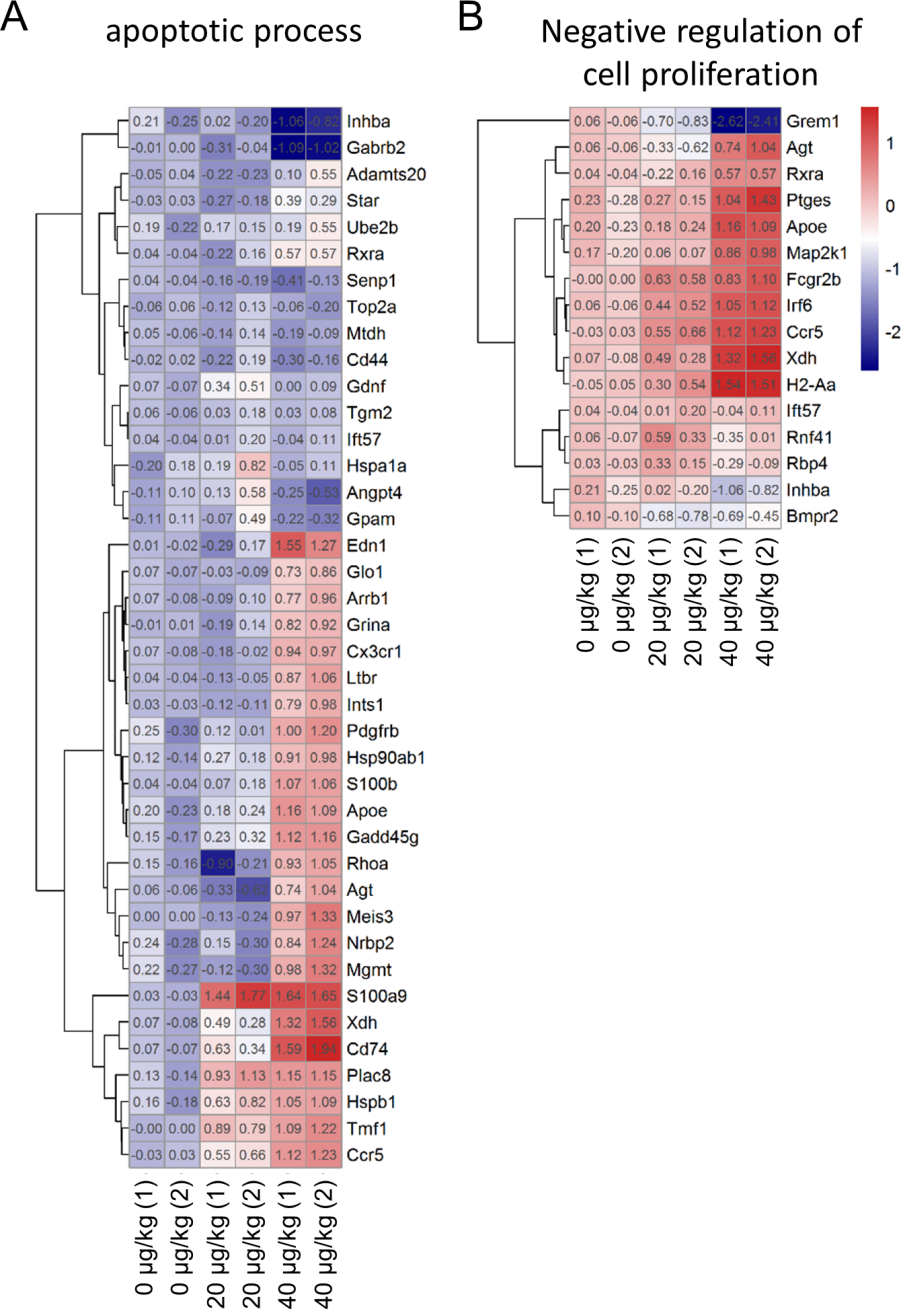
Heatmaps of DEGs. (A) apoptotic processes and (B) negative regulation of cell proliferation related genes expressions, via microarray, in control and DEHP-treatment groups.

After screened for differentially expressed genes (DEGs), we used DAVID to perform Gene Ontology enrichment analysis of the DEGs. We focused on genes involved in the apoptotic process and cellular proliferation to try to explain why DEHP caused an increase in the number of apoptotic ovarian somatic cells and to explain how DEHP inhibited antral follicle growth. We found that, compared with the control group, apoptosis related genes such as *Edn1*, *Pdgfrb*, *S100b*, *Apoe*, *Gadd45g*, *S100a9*, *Xdh*, *Cd74*, *Plac8*, *Hspb1*, *Tmf1* and *Ccr5* were significantly up-regulated in the 40 μg/Kg DEHP-treatment group (Fig 3A). The cell proliferation inhibition related genes such as *Apoe*, *Irf6*, *Ccr5*, *Xdh* and *H2-Aa* were also significantly up-regulated in the DEHP-treatment groups (Fig 3B). These results may provide evidence of the mechanism through which DEHP affects folliculogenesis.

**Fig 4:**
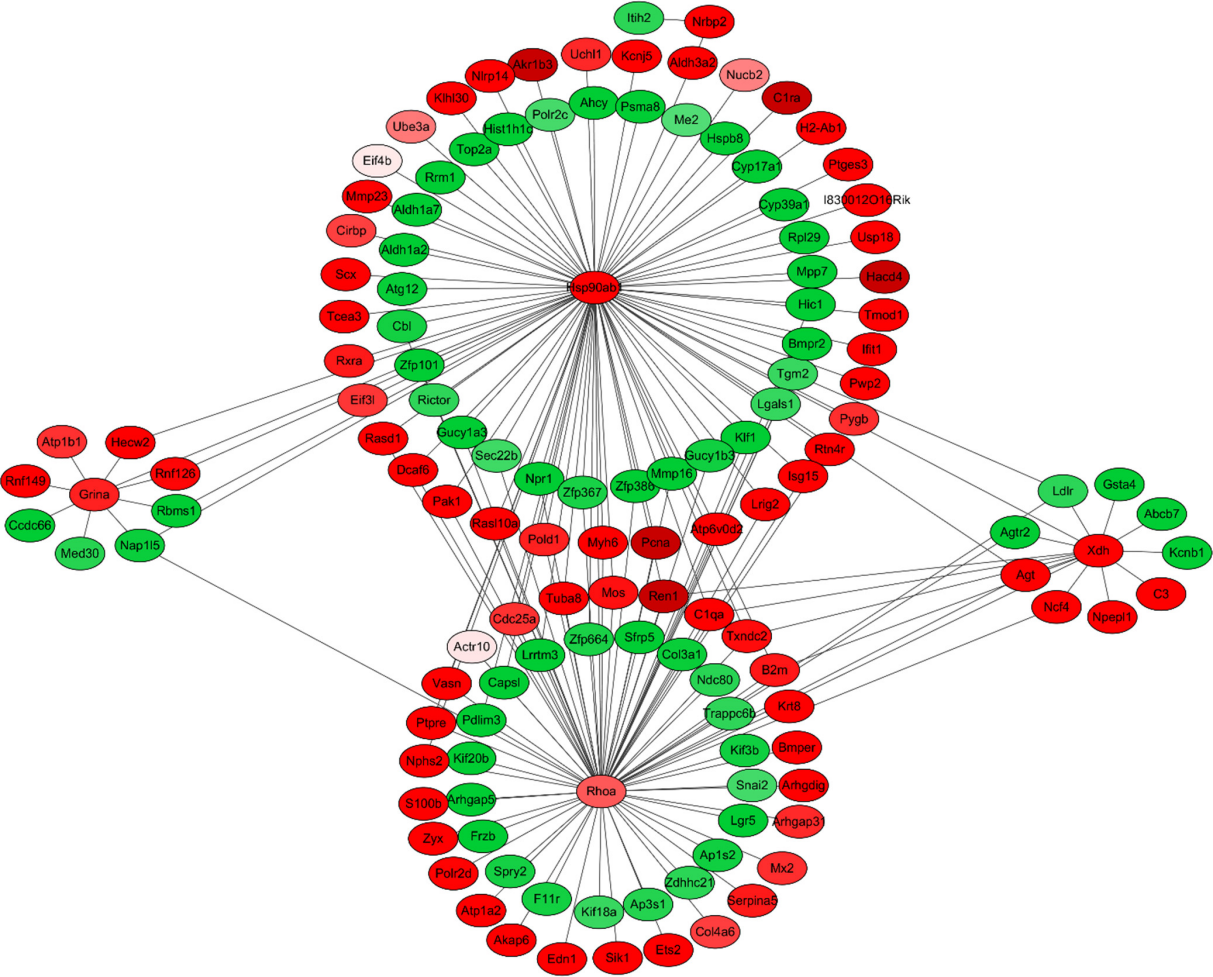
Protein interaction networks encoded by DEGs. Red points represent genes up-regulated and green points represent genes down-regulated in DEHP-treated groups.

Next, we screened the apoptosis related DEGs and used them to construct a protein interaction network. In the network, we found that *Hsp90ab1*, *Rhoa*, *Grina* and *Xdh* were node locations (Fig 4). Those four apoptosis related proteins directly or indirectly interacted with many other DEGs and regulated their functions.

**Fig 5:**
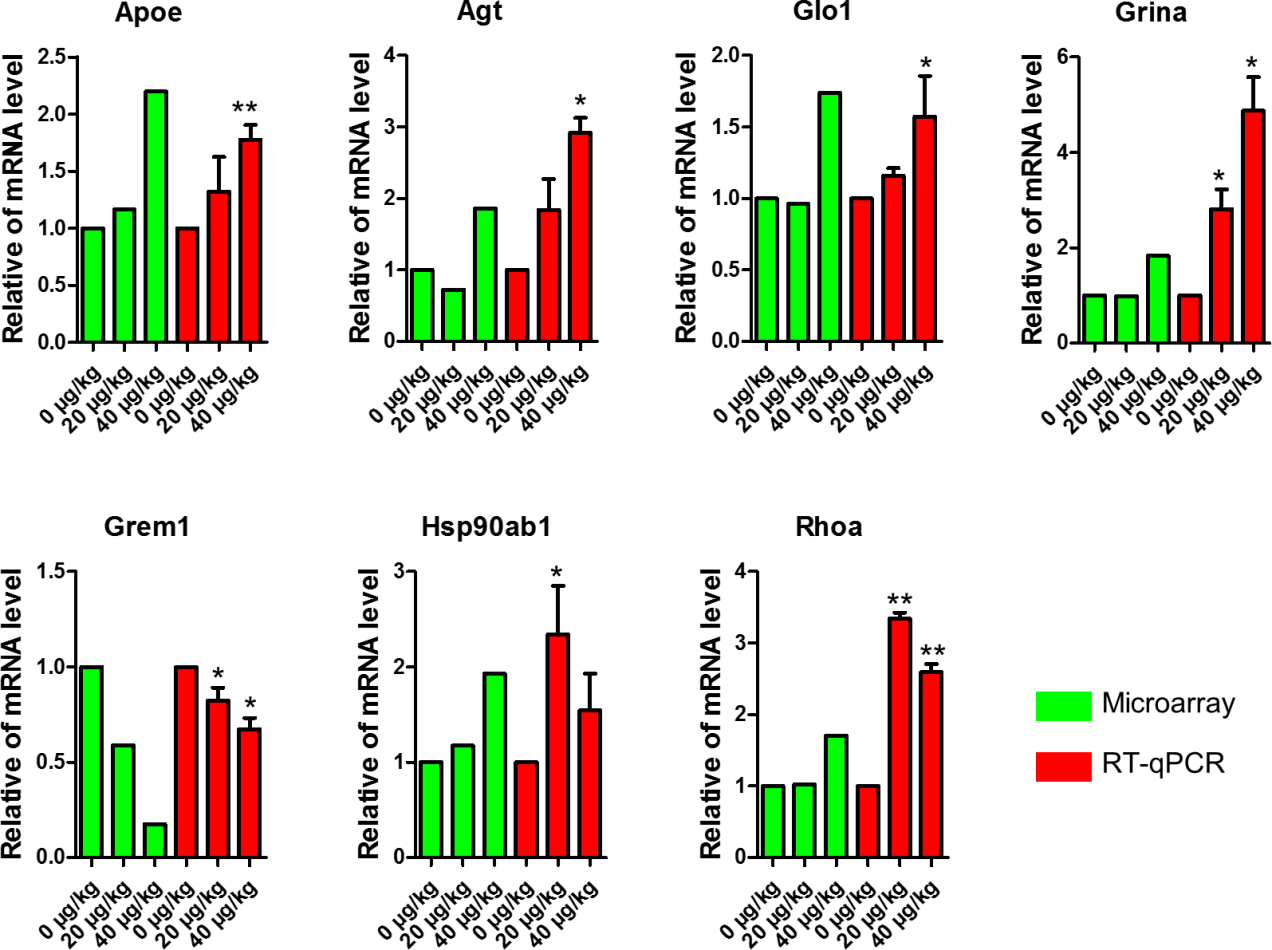
Comparison of qPCR data with that of microarray analysis. The green bars represent gene expression quantities of control and DEHP-treatment groups microarray data, and the red bars represent qPCR data in DEHP-treatment groups compared with the control group. Compared to the control group, relative fold changes were presented as mean ± SD. All experiments were repeated at least three times independently. (* P < 0.05; ** P < 0.01).

In order to validate the results of the microarrays we performed real-time qPCR to evaluate 7 DEGs of apoptosis and proliferation related genes. The results showed that the mRNA expression levels of *Apoe*, *Agt*, *Glo1*, *Grina*, *Grem1*, *Hsp90ab1* and *Rhoa* displayed a similar trend in both real-time qPCR and microarray (Fig 5). This may further explain how DEHP affects folliculogenesis via changes gene expression.

### DEHP Exposure Increases DNA Damage and Apoptosis of Ovarian Somatic Cells

**Fig 6:**
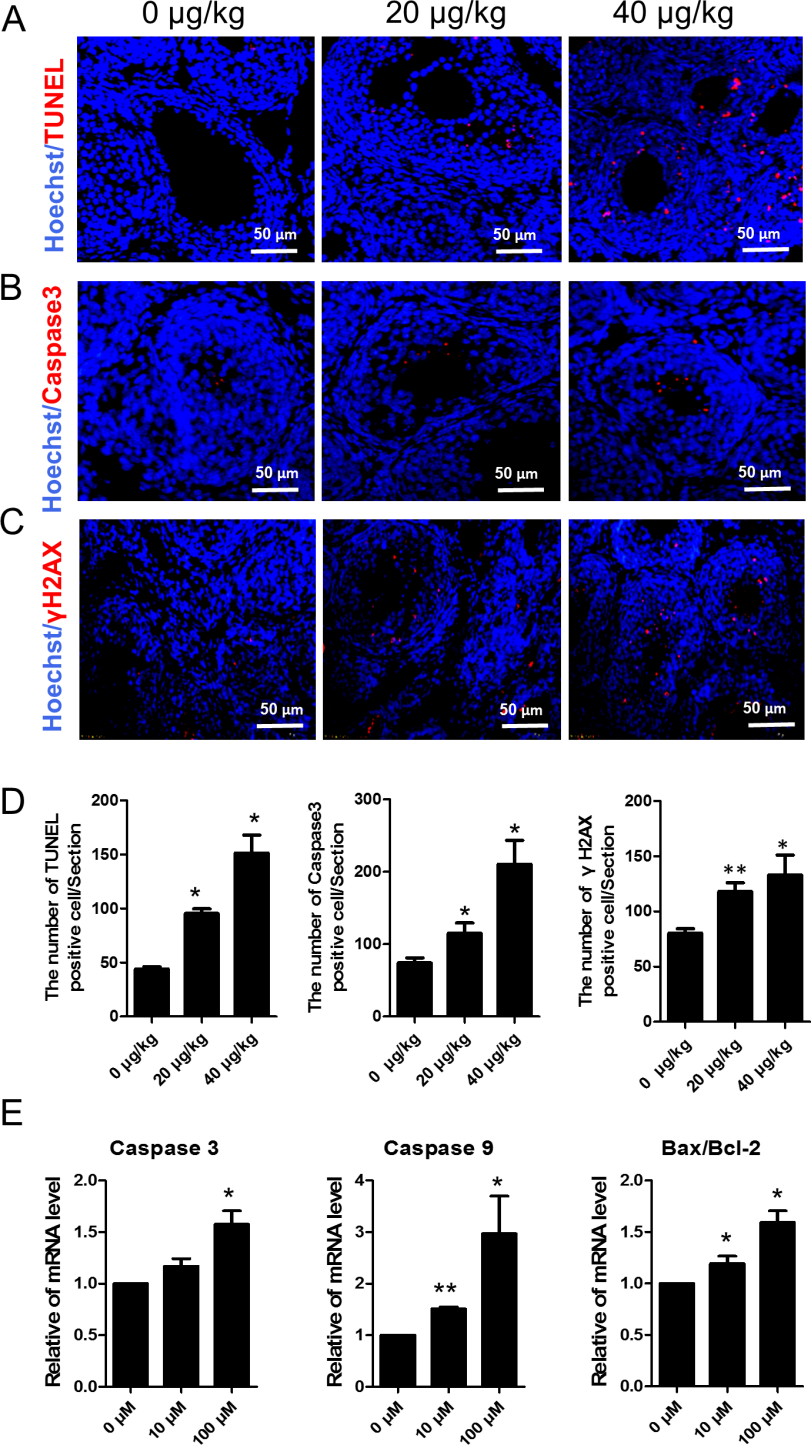
DEHP exposure increases DNA damage and apoptosis of ovarian somatic cells. (A) Apoptosis was examined by the terminal deoxyribonucleotidyl transferase-mediated dUTP-digoxigenin nick end-labelling (TUNEL) assay. The number of apoptotic cells increased significantly after treatment with DEHP when compared with the control group. (B) Apoptosis related proteins Caspase3 immunofluorescence. (C) DNA damage related proteins γH2AX immunofluorescence. Cell nucleuses are stained blue with Hoechst33342. (D) The numbers of TUNEL, Caspase3 and γH2AX positive cells per section. (E) The change of mRNA levels of related apoptosis genes *Caspase3*, *Caspase9*, and *Bax/Bcl-2* in the control and DEHP-treatment groups in *in vitro* experiments. Compared to the control group, relative fold changes were presented as mean ± SD. All experiments were repeated at least three times independently. (* P < 0.05; ** P < 0.01).

Since DEHP significantly inhibited large antral follicle formation, we investigated the effects of DEHP exposure on DNA damage and apoptosis in ovarian somatic cells. We performed TUNEL and immunofluorescence of the apoptosis protein Caspase3 and the double stranded breaks (DSBs) marker γH2AX in the DEHP treated mouse ovaries. The results show that TUNEL positive cells were mainly located in the ovarian somatic cells. Compared with the control the number of apoptotic cells were significantly increased in the DEHP-treatment groups (0 μg/kg = 44.0, 20 μg/kg = 95.3, 40 μg/kg = 151.3; P < 0.05) (Fig 6A and 6D). Immunohistochemistry results also showed that the number of Caspase3 positive cells in the 20 and 40 μg/kg DEHP-treatment groups is significantly higher than that of the control group (0 μg/kg = 74.0, 20 μg/kg = 115.0, 40 μg/kg = 210.3; P < 0.05) (Fig 6B and 6D). Similarly, the number of γH2AX positive cells (marker for DNA damage) in the 20 and 40 μg/kg DEHP-treatment groups is significantly higher than that of the control group (0 μg/kg = 80.3, 20 μg/kg = 118.0, 40 μg/kg = 133.0; P < 0.01 or P < 0.05) (Fig 6C and 6D) These results show that DEHP induced DNA damage and apoptosis in ovarian somatic cells. Furthermore, the mRNA level of apoptotic genes like *Caspase3*, *Caspase9* and *Bax*/*Bcl-2* was higher in the DEHP-treatment groups (Fig 6E).

### DEHP Exposure Induced Oxidative Stress Disorder in Ovarian Somatic Cells

In order to explore the mechanism of DEHP exposure induced apoptosis of ovarian somatic cells we detected the effects of DEHP exposure on the oxidative stress of the ovarian somatic cells both *in vivo* and *in vitro*.

**Fig 7:**
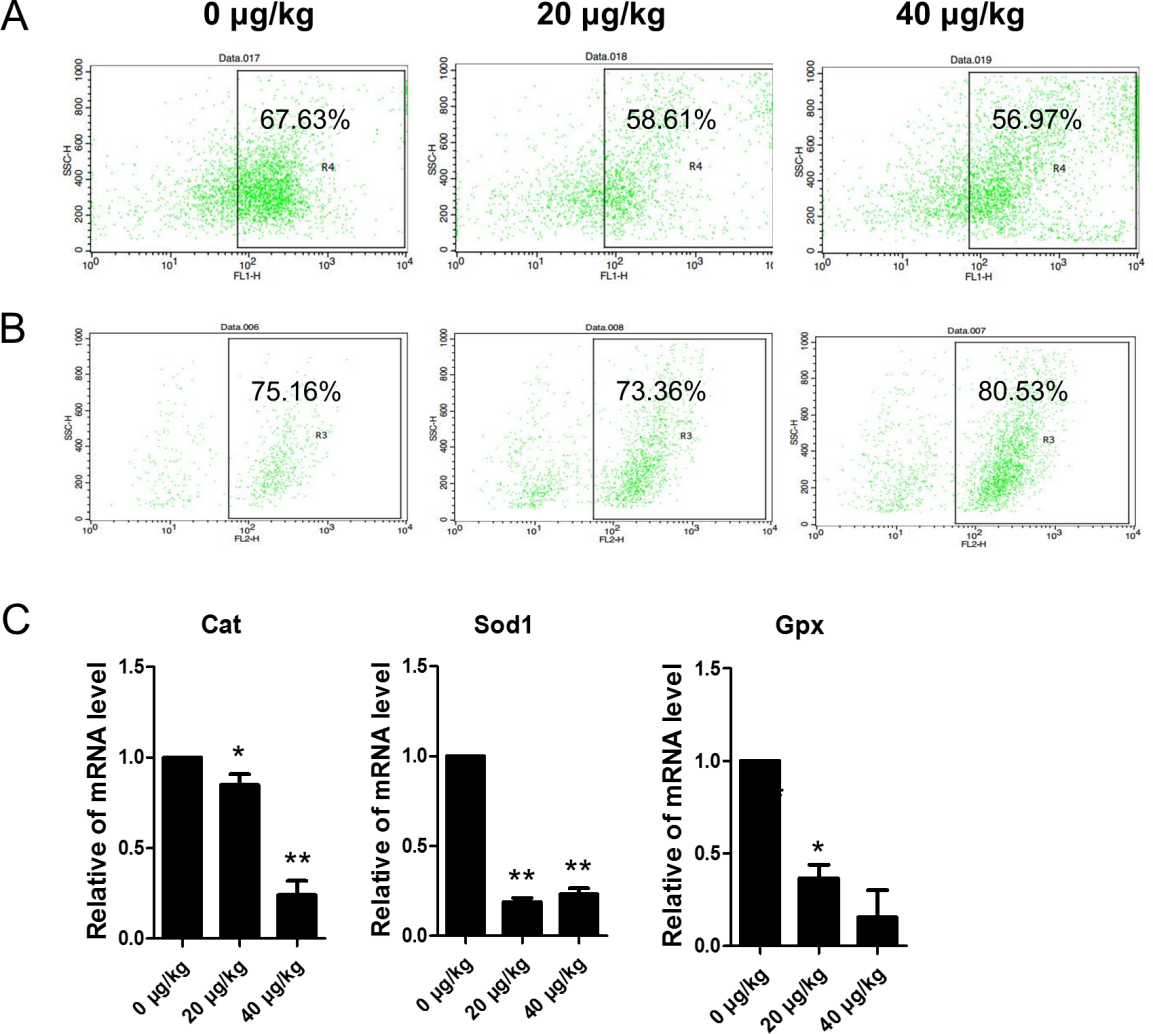
Effects of DEHP exposure on oxidative stress (DCF and DHE) and apoptosis in ovarian somatic cells. (A-B) Flow cytometer analysis of the oxidative stress in (DCF and DHE) ovarian somatic cells *in vivo*. (C) The change of mRNA levels of related oxidative stress genes *Cat*, *Sod1* and *Gpx* in the control and DEHP-treatment groups in *in vivo* experiments, respectively. Compared to the control group, relative fold changes were presented as mean ± SD. All experiments were repeated at least three times independently. (* P < 0.05; ** P < 0.01).

Flow cytometer analyses indicted that *in vivo* the level of DCF fluorescence decreased in the 20 or 40 μg/kg DEHP-treatment groups (58.61% or 56.97%) when compared with the control group (67.63%)(Fig 7A). However, the percentage of DHE positive ovarian somatic cells increased from 75.16% in the control group to 80.53% in the 40 μg/kg DEHP group (Fig 7B). As shown in Fig 7C, from the *in vivo* experiments, in the DEHP-treatment groups the mRNA levels of *Cat*, *Sod1* and *Gpx* were significantly (P < 0.05) or very significantly (P < 0.01) lower when compared to the controls.

**Fig 8:**
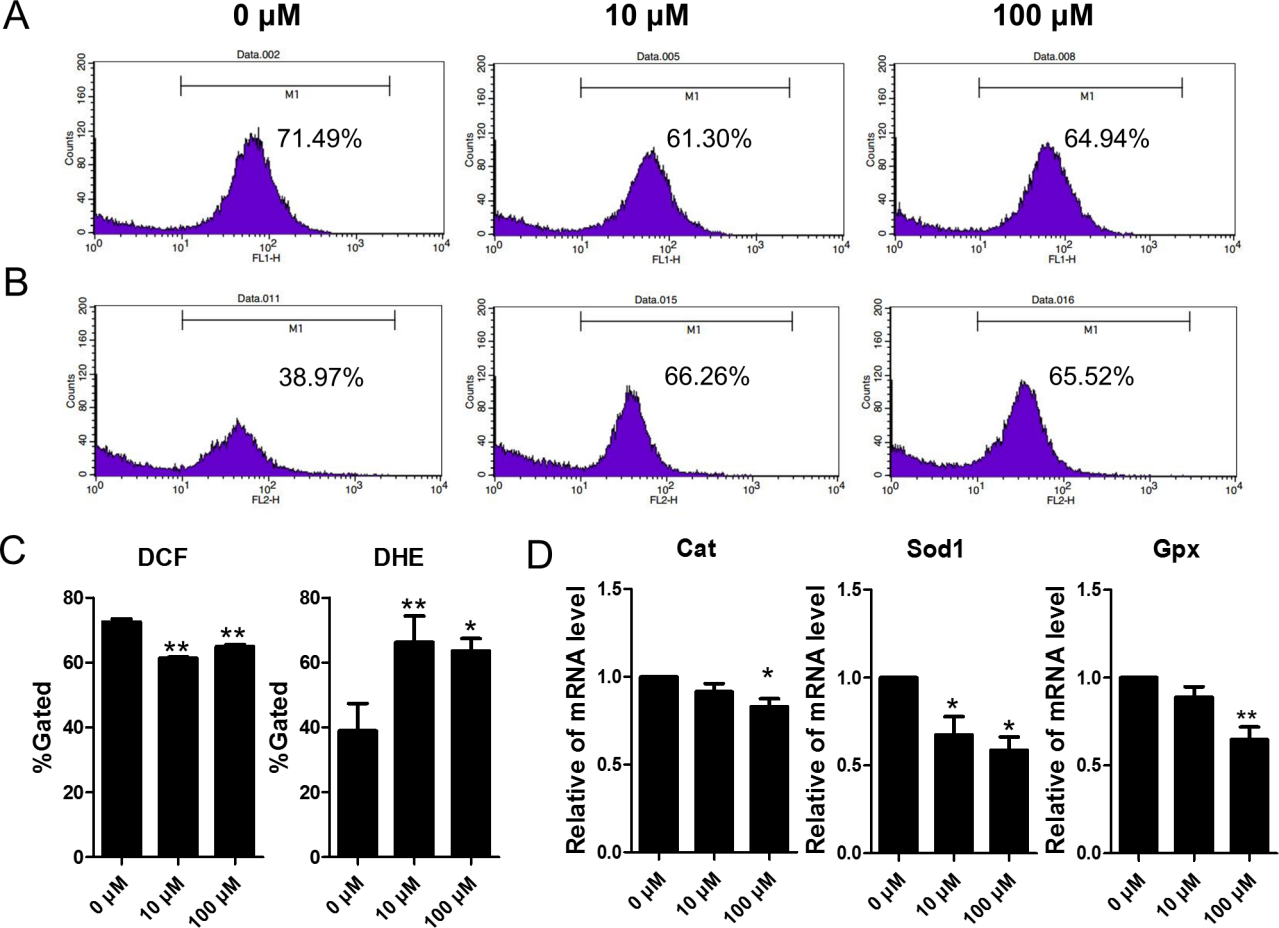
Effects of DEHP exposure on oxidative stress (DCF and DHE) and apoptosis in granulosa cells. (A-B) Flow cytometer analysis of the oxidative stress in (DCF and DHE) ovarian somatic cells *in vitro*. (C) Percentages of DCF and DHE positive cells in each group. (D)The change of mRNA levels of related oxidative stress genes *Cat*, *Sod1* and *Gpx* in the control and DEHP-treatment groups in *in vivo* experiments, respectively. Compared to the control group, relative fold changes were presented as mean ± SD. All experiments were repeated at least three times independently. (* P < 0.05; ** P < 0.01).

To test the *in vivo* results, *in vitro* experiments using cultured ovarian somatic cells exposure to DEHP were utilized. As shown in Fig 8A–8C, the level of DCF decreased and DHE increased significantly (P < 0.05) or very significantly (P < 0.01) in DEHP-treatment groups, *in vitro*. Also, the mRNA levels of *Cat*, *Sod1* and *Gpx* were significantly (P < 0.05) or very significantly (P < 0.01) lower in the DEHP-treatment groups when compared to the control groups (Fig 8D).

## Discussion

DEHP has proven to be a reproductive and ovarian function disrupter that impacts mouse follicular development [10, 24, 29, 34]. In our study, we demonstrated the effects of DEHP on pre-pubertal mouse ovarian follicle development. Specifically, we confirmed that DEHP inhibited large antral follicle formation. In addition, DEHP induced DNA damage and apoptosis of the ovarian somatic cells, disrupting the ovarian oxidative status.

Previous studies demonstrated that DEHP is an endocrine-disrupter and has reproductive toxic effects. Hannon and his colleagues reported that DEHP interferes with PI3K signaling and can induce an acceleration of primordial follicle development [20]. Also, an *in vitro* study demonstrated that DEHP affected oocyte maturation and the embryogenesis process [35]. Our previously work also indicated that DEHP induced sexual precocity, abnormal oocyte development, and premature ovarian failure [24]. Additionally, when pregnant mice were exposed to DEHP, the expression of the germ cell markers *Stra8*, *Dazl*, and *Nobox* in fetal mice were all down regulated and the fetal oogenesis processes were delayed [25]. All these data demonstrate that DEHP has wide reproductive toxic effects at different development stages in female mice. However, the effects of DEHP on prepubescent mouse ovarian development, particularly folliculogenesis, are still poor understood. An *in vitro* study has shown that DEHP impacts early folliculogenesis, they focused on the stage of neonatal mouse ovarian germ cell nest breakdown and primordial follicle formation [29]. Although Hannon et al. have reported that MEHP rather than DEHP directly inhibits steroidogenesis and accelerates primordial follicle activation in postnatal day 4–10 *in vitro* culture [22], and MEHP inhibits antrum growth of tertiary follicles *in vitro*, the knowledge of DEHP effects on antral follicle formation in pre-pubertal mice *in vivo* is still incomplete. Also, MEHP has been shown to directly inhibit antral follicle enlargement during *in vitro* culture [10, 36]. In the present study, we found that the number of large antral follicles (>150 μm antrum size) per ovary in DEHP-treatment groups were significantly less than that seen in the control group. Thus, we designed the microarray experiments to investigate the effects of DEHP from 5 dpp to 20 dpp on ovarian gene expression *in vivo*. This period covers neonatal, juvenile, and pre-pubertal ovarian development, which includes primordial follicle development, along with primary, secondary and antral follicle growth. From our results, DEHP inhibited large antral follicle formation. This *in vivo* result indicated that DEHP inhibited the antral follicle enlarge process.

We assumed that, DEHP has a toxic effect and potentially affected ovarian somatic cell survival. Therefore, we assessed the oxidative stress, apoptosis, and DNA damage status of the ovarian somatic cells. The results showed that TUNEL positive cell numbers in DEHP-treatment groups were significantly higher than that in the control group. Additionally, the number of Caspase 3 positive cells in the DEHP-treatment groups were larger than that in the control group. These results indicated that DEHP increased follicle somatic cell apoptosis via increased Caspase 3 synthesis, which may explain how DEHP inhibits the antral follicle enlargement process of pre-pubertal mice. γH2AX is generally thought to be a predominant protagonist in DNA double-strand-breaks (DSB) [37] which has the main role of recruiting DNA repair proteins such as RAD51 [38]. Several key proteins intersect the DNA repair pathway with the apoptotic pathways such as *P53*, *BRCA1*, *ATM* and others [39]. In our study, compared with the control group, the number of γH2AX positive ovarian somatic cells in the 20 and 40 μg/kg DEHP treatment groups was significantly higher. These results indicate that DEHP induces more DSB foci in ovarian somatic cells and may initiate somatic cells to enter apoptotic pathways. Further study to investigate the relationship between DEHP induced ovarian somatic cells apoptosis and DNA damage is needed.

Previous studies reported that DEHP could significantly decrease pre-pubertal SD-rat testicular antioxidative ability [9]. In female MEHP can increase ROS levels and inhibit the expression and activities of *Sod1* and *Gpx*, but not *Cat* [40]. While DEHP induced production of ROS and decreased the expression and activity of *Sod1* [40]. In our study, the expression of *Sod1*, *Gpx* and *Cat* were all decreased after exposure to DEHP, *in vivo*. Also, in the *in vitro* DEHP-treatment group the expression of *Sod1*, *Gpx*, and *Cat* were decreased. DEHP induced an increase of ROS both *in vivo* and *in vitro*. Previous studies have shown that oxidative stress affected folliculogenesis, endometriosis, oocyte maturation and fertilization [4, 41–43]. Endocrine disrupting chemicals such as BPA, Zearalenone (ZEA), and DEHP caused oxidative stress in mammalian ovaries [44–46], and our data shows that DEHP induced oxidative stress and could impact ovarian function. In our present study DEHP inhibited *Sod1*, *Gpx*, and *Cat* expression both *in vivo* and *in vitro*. In addition, DEHP decrease the H_2_O_2_ levels but significantly induced super oxide anion accumulation in ovarian somatic cells, *in vitro*. This indicated that DEHP directly disturbs the oxidative status of ovarian somatic cells.

In our study, to investigate the mechanism of DEHP effects on ovarian development of prepuberty mice we performed microarray experiments. Via microarray we have demonstrated that exposure of 20 μg/kg and 40 μg/kg DEHP altered ovarian gene expression patterns. Compared with the control group the expression of apoptosis related genes and cell proliferation related genes were changed as a whole. The apoptosis related genes *Edn1*, *Pdgfrb*, *S100b*, *Apoe*, *Gadd45g*, *S100a9*, *Xdh*, *Cd74*, *Plac8*, *Hspb1*, *Tmf1* and *Ccr5* were significantly up-regulated in the 40 μg/kg DEHP-treatment group. Cell proliferation inhibition related genes *Apoe*, *Irf6*, *Ccr5*, *Xdh* and *H2-Aa* were also significantly up-regulated. Next, we screened the apoptosis related DEGs whether in the 20 or 40 μg/kg groups. We used their coded proteins and constructed a protein interaction network. In the network, we found that *Hsp90ab*, *Rhoa*, *Grina* and *Xdh* were node locations. Those four apoptosis related proteins directly or indirectly interacted with many other DEGs encoded proteins, and thereby regulated their functions. We verified the expression of a subset of the apoptosis and proliferation related genes by qPCR. We found the expression trend of the tested genes, such as *Apoe*, *Glo1*, *Grina* and *Grem1* in qPCR was similarly to that of microarray. This indicated that DEHP most likely affected folliculogenesis through the above molecules. Heat shock proteins (*Hsps*) are molecular chaperones which help protein folding and protect target proteins during stressful conditions [47]. From previous research, compared with *in vivo*-matured cumulus oocyte complexes (COCs), *in vitro* maturation led to an increase of apoptosis and heat shock-related genes including *Hsp90ab1* [48]. In our study, the results of qPCR and microarray showed that DEHP increased the expression of *Hsp90ab1*. This indicated that DEHP induced follicles suffered stress conditions that may well be caused by oxidative stress. In addition, a previous study demonstrated that *Rhoa* activation induces the enrichment of integrin in the surface of cumulus cells, and then remodels the extracellular matrix [49]. This process would inhibit the fertilization process. In our present and previous results DEHP induced increased expression of *Rhoa* in prepuberty mice and reduced the litter size of female mice. Therefore the relationship between DEHP and fertilization and cumulus cells expansion requires further study.

Taken together, our data demonstrated that DEHP inhibited antral follicle enlargement during the pre-pubescent stages. This inhibition may be mediated by increasing oxidative stress and ovarian somatic cell apoptosis. These results suggest that DEHP exposure could be dangerous for pre-pubescent ovarian follicle development.
